# Crotonoside exhibits selective post-inhibition effect in AML cells via inhibition of FLT3 and HDAC3/6

**DOI:** 10.18632/oncotarget.20710

**Published:** 2017-09-08

**Authors:** Yu-Zhi Li, Si Yu, Pei-Ao Yan, Dao-Yin Gong, Fang-Li Wu, Zhi He, Yu-Yao Yuan, An-Yan Zhao, Xue Tang, Ruo-Qi Zhang, Cheng Peng, Zhi-Xing Cao

**Affiliations:** ^1^ Pharmacy College, Chengdu University of Traditional Chinese Medicine, The Ministry of Education Key Laboratory of Standardization of Chinese Herbal Medicine, Key Laboratory of Systematic Research, Development and Utilization of Chinese Medicine Resources in Sichuan Province-Key Laboratory Breeding Base of Co-founded by Sichuan Province and MOST, Sichuan, China; ^2^ Second Clinical Medical College, Chengdu University of Traditional Chinese Medicine, Sichuan, China

**Keywords:** AML, crotonoside, FLT3, HDAC3, HDAC6

## Abstract

Targeted therapies for the treatment of acute myeloid leukemia (AML), specifically the FLT3 inhibitors, have shown promising results. Nevertheless, it is very unlikely that inhibitors which target a single pathway will provide long-term disease control. Here, we report the characterization of crotonoside, a natural product extracted from Chinese medicinal herb, Croton, for the treatment of AML via inhibition of FLT3 and HDAC3/6. *In vitro*, crotonoside exhibited selective inhibition in AML cells. *In vivo*, crotonoside treatment at 70 and 35 mg/kg/d produced significant AML tumor inhibition rates of 93.5% and 73.6%, respectively. Studies on the anti-AML mechanism of crotonoside demonstrated a significant inhibition of FLT3 signaling, cell cycle arrest in G0/G1 phase, and apoptosis. In contrast to classic FLT3 inhibitor; sunitinib, crotonoside was able to selectively suppress the expression of HDAC3 and HDAC6 without altering the expression of other HDAC isoforms. Inhibitors of HDAC3 and HDAC6; RGFP966 and HPOB, respectively, also exhibited selective inhibition in AML cells. Furthermore, we established novel signaling pathways including HDAC3/NF-κB-p65 and HDAC6/c-Myc besides FLT3/c-Myc which are aberrantly regulated in the progression of AML. In addition, crotonoside alone or the combination of sunitinib/RFP966/HPOB exhibited a significant post-inhibition effect in AML cells by the inhibition of FLT3 and HDAC3/6. Inhibitors targeting the FLT3 and HDAC3/6 might provide a more effective treatment strategy for AML. Taken together, the present study suggests that crotonoside could be a promising candidate for the treatment of AML, and deserves further investigations.

## INTRODUCTION

Acute myeloid leukemia (AML) is a clinically and biologically heterogeneous hematological disorder with high mortality rate [1-3]. The genetic variations contribute to the occurrence and development of AML, mutations in the receptor tyrosine kinase FMS-Like Tyrosine Kinase-3 (FLT3) are present in about one-third of all the patients with AML [4, 5]. In several clinical trials, FLT3 inhibitors could induce complete or partial remissions in the majority of patients with AML, however, drug resistance is frequently encountered within several months of treatment [6, 7]. Consequently, several studies suggested that other signaling pathways might contribute to the transformed drug-resistant phenotype, and thus these pathways besides FLT3 can be exploited for pronounced therapeutic benefits [8].

FLT3 encodes a membrane-bound receptor tyrosine kinase (RTK) which belongs to the RTK subclass III family. FLT3 is normally expressed by primitive hematopoietic progenitor cells within the bone marrow [9, 10]. FLT3 activating mutations normally occur in the AML and rarely in the ALL. FLT3 internal tandem duplication (FLT3-ITD) is the most prevalent activating mutation and associated with a very poor prognosis in AML patients [11-13]. Several small-molecule FLT3 inhibitors, including Sunitinib, Ponatinib, PKC-412, Sorafenib, and AC220, are being evaluated in the recent clinical trials, showed promising anti-tumor activity [14-18]. However, the clinical effectiveness of FLT3 inhibitors in patients with AML was quite variable and, exhibited the modest clinical activity, and the clearance of peripheral leukemia blast cells. All responses were relatively transient, lasting weeks to months. Progressive disease, relapse or drug resistance often occurs within short duration [19-21]. The causes of relapse and drug resistances are complex. More recently, single cell analysis demonstrated the existence of FLT3-ITD negative cells in clinically determined FLT3-ITD positive patients indicated the intra-tumor heterogeneity. Furthermore, multiple mutations are also the most important contributors in the relapse and FLT3 inhibitor resistance [22, 23].

HDACs (Histone deacetylases) are enzymes that catalyze the removal of acetyl functional groups from both histone and non-histone proteins as well as many other nuclear, mitochondrial, and cytoplasmic proteins [24, 25]. In AML, HDACs exhibit a distinctive pattern of expression and play a key role in regulating gene expression by changing chromatin structure [26, 27]. Inhibition of HDACs by drugs such as vorinostat or depsipeptide has emerged as an effective treatment strategy in AML [28-30]. However, despite their promising activity in pre-clinical studies, HDAC inhibitors have demonstrated only modest anti-tumor activity in patients with AML. Further, the toxicity profiles of non-selective HDAC inhibitors in the combination settings also limit the clinical benefit [31-33]. Thus, the development of novel isoform-selective HDAC inhibitor in combination with other inhibitors is urgently required to exploit the therapeutic potential of HDAC inhibitors.

Recently, Wang and Armstrong have demonstrated that specific inhibition of HDAC3 or HDAC6 could prove useful for the treatment of AMLs [34, 35]. We hypothesized that simultaneous inhibition of FLT3 and the HDAC3/6 may provide greater therapeutic benefits in the treatment of AML. Therefore, the present study identified and characterized crotonoside a natural product extracted from Chinese medicinal herb of Croton that could inhibit the activation of FLT3 and decrease the expression of HDAC3/6. In conclusion, crotonoside exhibited selective inhibition in AML cells *in vitro* and suppressed the growth of AML cell with well tolerance *in vivo*.

## RESULTS

### *In vitro* growth-inhibitory effects of crotonoside against leukemia and other cell lines

**Figure 1:**
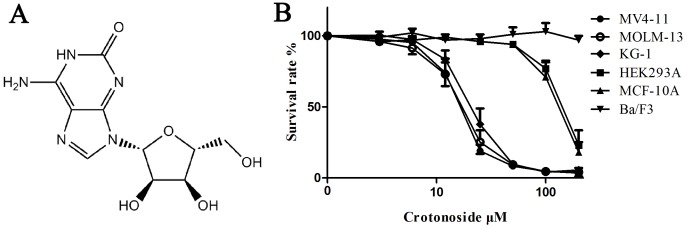
Effects of crotonoside on the viability of AML cells and normal cell lines **(A)** The chemical structure of crotonoside. **(B)** AML cells or normal cells lines treated with different concentrations of crotonoside (0-200 μM) for 72h. Cell viability was measured by MTT assay. Data are representative of more than three independent experiments.

The *in vitro* growth inhibitory activities of crotonoside were evaluated against a panel of cell lines, including leukemia and solid tumor cell lines. As presented in Table 1, crotonoside selectively inhibited the viability of AML cell line MV4-11, MOLM-13 (with FLT3-ITD mutant) and KG-1 (without FLT3-ITD mutant) in a dose-dependent manner with an IC50 of 11.6±2.7 μM, 12.7±3.3 μM and 17.2±4.6 μM, respectively. However, a relatively decreased cytotoxicity was observed in normal human embryonic kidney cell (HEK293A; IC50: 182.8±34.9 μM), human mammary epithelial cell line (MCF-10A; IC50: 167.3±38.3 μM) and several other cancer cell lines, including A549, MDA-MB-231. Negligible activity was observed against the murine pro-B cell line Ba/F3 and remaining 12 human cancer cell lines (Table 1 and Figure 1). These results indicated that AML cells were highly sensitive to crotonoside than other cell lines tested.

**Table 1:** Antiproliferative profile of crotonoside on various cell lines.

Cell line	Cell type	IC50 (μM)
MV4-11	Human acute myeloid leukemia	11.6±2.7
KG-1	Human acute myeloid leukemia	17.2±4.6
MOLM-13	Human acute myeloid leukemia	12.7±3.3
A549	Human lung cancer	165.6±31.6
MCF-10A	Human breast cancer	167.3±38.3
MDA-MB-231	Human breast cancer	176.8±29.2
HEK293A	Human embryonic kidney cell	182.8±34.9
Ba/F3	Murine pro-B cell line	>200
Hela	Human epitheloid cervix carcinoma	>200
SUDHL-6	Human B cell lymphoma	>200
HBL-1	Human B cell lymphoma	>200
Ramos	Human Burkitt lymphoma	>200
Raji	Human Burkitt lymphoma	>200
Jurkat	Human acute T cell leukemia	>200
HCC827	Human lung adenocarcinoma	>200
Huh-7	Human hepatocellular carcinoma	>200
PLC/PRF/5	Human hepatocellular carcinoma	>200
A2058	Human melanoma	>200
UACC-62	Human melanoma	>200
Miapaca-2	Human pancreatic cancer	>200

### Multiple signaling inhibitions by crotonoside in MV4-11 cells

**Figure 2:**
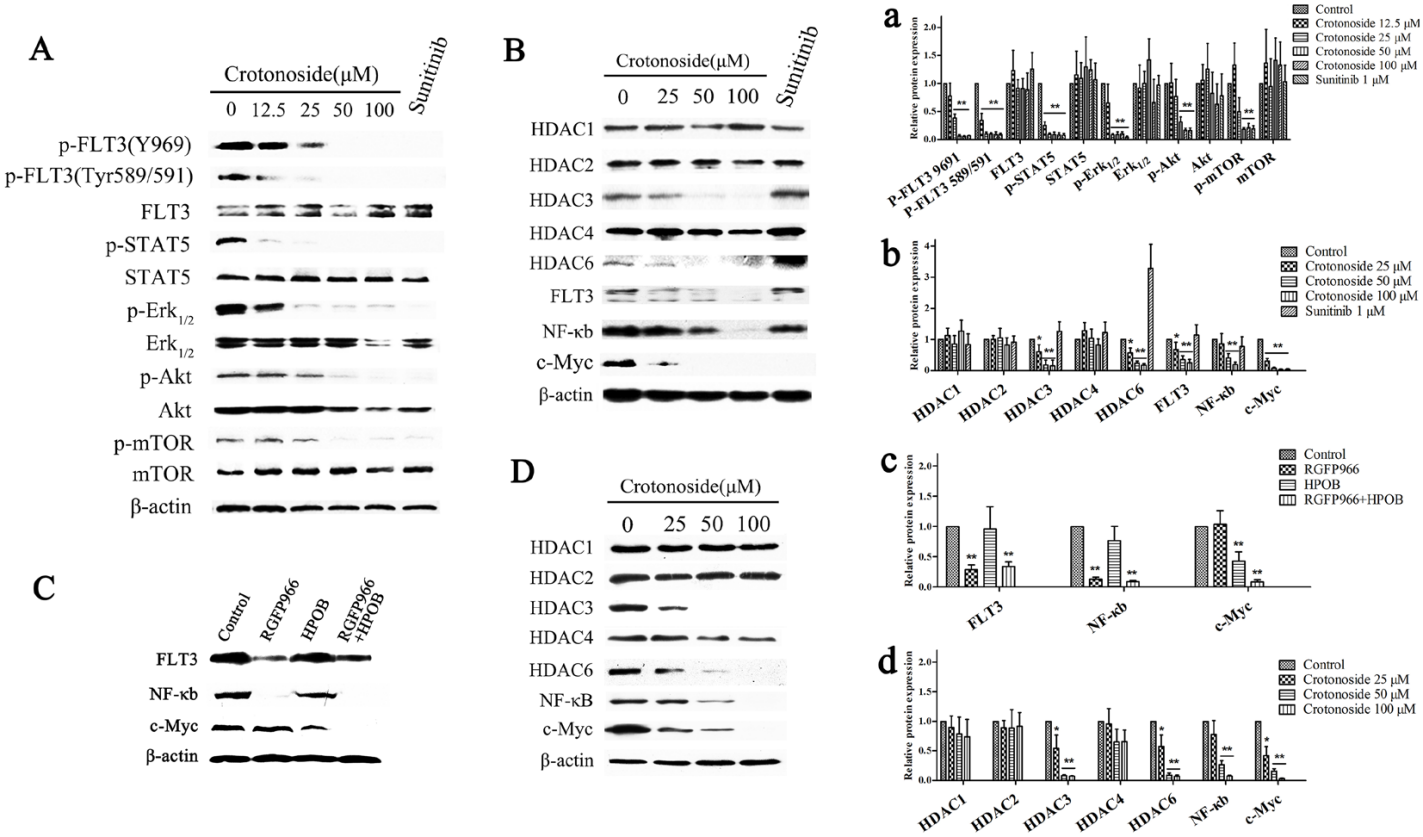
Crotonoside inhibited the activation of FLT3 signal and decreased the expressions of HDAC3 and HDAC6 **(A)** MV4-11 cells were treated with different concentrations of crotonoside or 0.1% DMSO for 7 h, 1 μM sunitinib was considered as positive control. The phosphorylation of FLT3 and downstream signal proteins was assessed using Western blot assay. **(B)** Western blot analysis of the expression level of HDACs, FLT3, NF-κB-p65, and c-Myc in MV4-11 cells treated with crotonoside or sunitinib for 20 h. **(C)** Western blot analysis of the expression of FLT3, NF-κB-p65, and c-Myc in MV4-11 cells treated with control, 10 μM RGFP966 (HDAC3-selective inhibitor), 20 μM HPOB (HDAC6-selective inhibitor), or in the combination of RGFP966 and HPOB for 24 h. **(D)** Western blot analysis of the expression of HDACs、NF-κB-p65 and c-Myc in KG-1 cells treated with crotonoside for 20 h. a-d, the relative protein expression were analyzed using image analyzing software. Statistical analysis was performed to analyze the differences (a for A, b for B, c for C, d for D; ^*^, *p*<0.05; ^**^, *p*<0.01).

The ability of crotonoside to inhibit the important signaling pathways in MV4-11 cells was analyzed using western blot assay (Figure 2A). Our results showed that the incubation of MV4-11 cells with crotonoside (7 h) inhibited the phosphorylation of FLT3 in a dose-dependent manner. The activation of downstream signaling proteins Erk1/2, Akt/mTOR and STAT5 was also strongly inhibited by crotonoside at higher concentration of 12.5 μM in a concentration-dependent manner. Similarly, positive control, sunitinib also significantly inhibited the phosphorylation of FLT3, Erk1/2, Akt/mTOR and STAT5 in MV4-11 cells (*p*<0.05). Moreover, crotonoside did not modulate the expression of these proteins during the treatment period.

Furthermore, crotonoside significantly inhibited the expression of FLT3 after 20 h of the treatment, so we performed western blot assay to analyze the additional signaling pathways which might be inhibited by crotonoside in MV4-11 cells. As illustrated in Figure 2B, crotonoside significantly inhibited the expression of HDAC3 and HDAC6 at higher concentrations of 25 μM (*p*<0.05), however, the expression of other isoforms, including HDAC1, HDAC2, and HDAC4 was not modulated by crotonoside. Sunitinib (1uM) exhibited similar inhibitory effect on the activation of FLT3 signal. However, the expression of HDAC3 was unaltered, and the expression of HDAC6 was even found to be upregulated. Furthermore, crotonoside also inhibited expression of transcription factors, NF-κB-p65, and c-Myc, while sunitinib only inhibited the expression of c-Myc.

We further investigated the effect of selective inhibition of HDAC3 or HDAC6 on the expression of FLT3 and the transcription factors. MV4-11 cells were treated with control or 10 μM RGFP966 (HDAC3-selective inhibitor), or 20 μM HPOB (HDAC6-selective inhibitor), or in combination. After 20 hours, cells were harvested for western blot analysis. The results from western blot analysis are shown in Figure 2C, RGFP966 significantly inhibited the expression of FLT3 and NF-κB-p65, HPOB significantly inhibited the expression of c-Myc, and the combination of RGFP966 and HPOB significantly inhibited the expression of FLT3, NF-κB-p65 and c-Myc (*p*<0.05). These results indicated that the inhibition of expression of FLT3 and NF-κB-p65 by crotonoside was due to the inhibition of the HDAC3, and the inhibition of c-Myc expression by crotonside was due to the inhibition of the FLT3 and HDAC6.

In addition, we also analyzed the anti-AML mechanisms of crotonoside in KG-1 cells. KG-1 cells were harvested and lysed for a western blot assay after 20 h of treatment with crotonoside. As illustrated in Figure 2D, crotonoside significantly inhibited the expression of HDAC3 and HDAC6 at a higher concentrations 25 μM (*p*<0.05), however, no effect was observed on the expression of other isoforms, HDAC1, HDAC2, and HDAC4. Interestingly, crotonoside also inhibited the expression of transcription factors, NF-κB-p65 and c-Myc in KG-1 cells.

### RGFP966 and HPOB exhibit selective inhibition in AML cells

**Figure 3:**
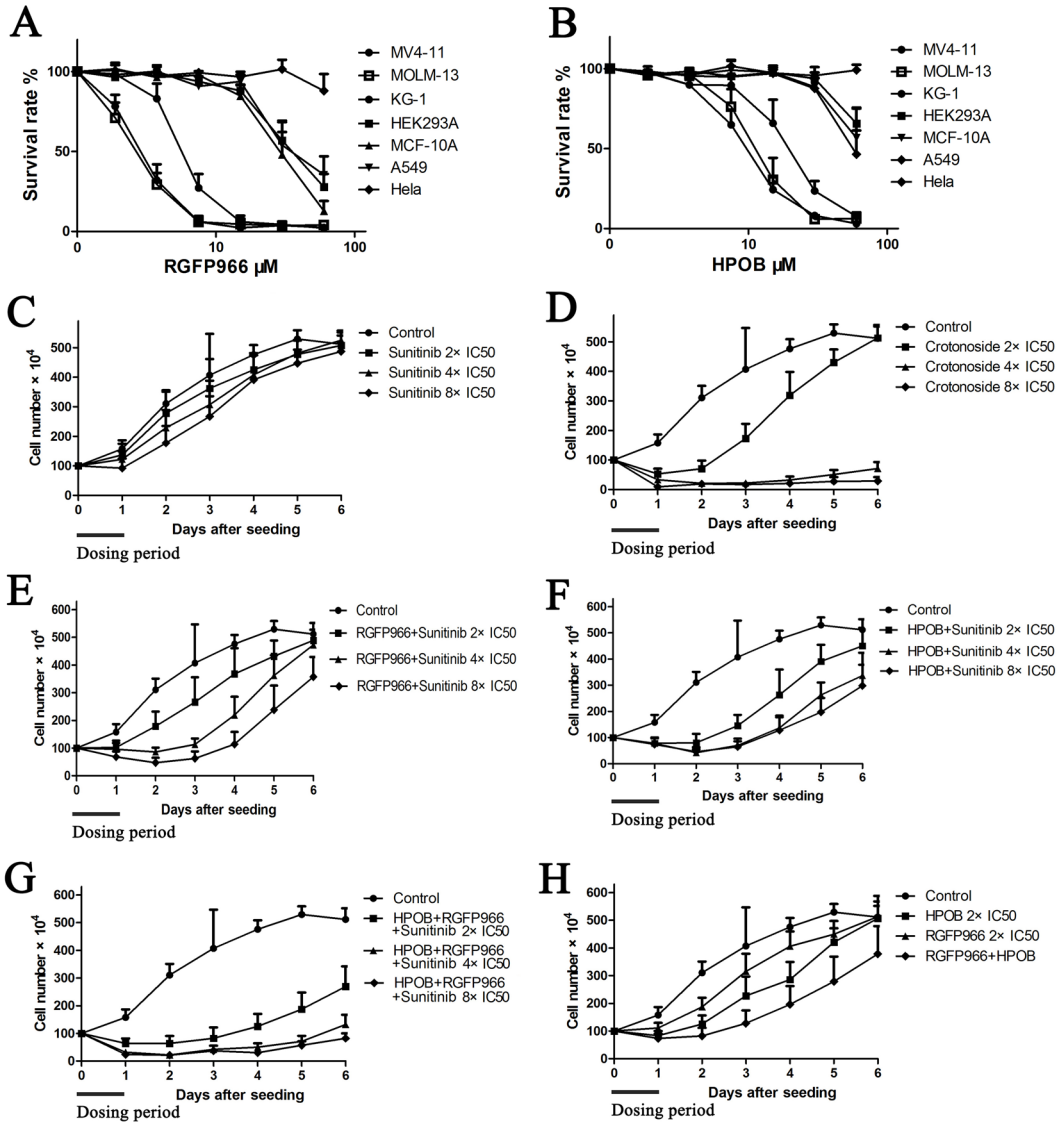
Crotonoside exhibit selective and efficient inhibition in AML cells compared to positive control, sunitinib via inhibition of FLT3 and HDAC3/6 The anti-viability activity of RGFP966 **(A)** and HPOB **(B)** against MV4-11 and other cell lines were assessed by MTT assay. Further, the post-inhibition effects of a single drug or in combinations were analyzed. Briefly, after being treated by a single drug or the combinations, MV4-11 cells were harvested and re-cultured in fresh medium and the cells’ growth viability was evaluated by counting the cell number daily for 6 days. **(C)** The post-inhibition effect of sunitinib against MV4-11 cells at the concentrations of 2×IC50, 4×IC50, and 8×IC50. **(D)** The post-inhibition effect of crotonoside against MV4-11 cells at the concentrations of 2×IC50, 4×IC50, and 8×IC50. **(E)** The post-inhibition effect of the combination of RGFP966 2×IC50 and sunitinib 2×IC50, 4×IC50 or 8×IC50. **(F)** The post-inhibition effect of the combination of HPOB 2×IC50 and sunitinib 2×IC50, 4×IC50 or 8×IC50. **(G)** The post-inhibition effect of the combination of HPOB 2×IC50, RGFP966 2×IC50 and sunitinib 2×IC50, 4×IC50 or 8×IC50. **(H)** The post-inhibition effect of HPOB 2×IC50, RGFP966 2×IC50 and their combination.

The growth inhibitory activity of RGFP966 and HPOB against AML and other cell lines were analyzed. As shown in Figure 3A, RGFP966 inhibited the viability of MV4-11, MOLM-13 and KG-1 with IC50 values of 3.6±0.7 μM, 2.8±0.9 μM and 6.91±2.2 μM. HPOB inhibited the viability of both MV4-11 and KG-1 with IC50 values of 10.2±2.9 μM, 13.6±5.2 μM and 21.49±7.7 μM, respectively. Nevertheless, RGFP966 and HPOB both exhibited lower inhibitory activities against normal human embryonic kidney cell HEK-293A, human mammary epithelial cell line MCF-10A, human lung cancer cell line A549, and human breast cancer cell line MDA-MB-231. These results indicated that the HDAC3-selective inhibitor and HDAC6-selective inhibitor both possessed selective inhibition pattern toward AML cell.

### Crotonoside exhibits post-inhibition effect in AML cell by the inhibition of FLT3 and HDAC3/6

To evaluate the significance of crotonoside in comparison to other selective inhibitors, we performed a cell growth assay to analyze the post-inhibition effect of these inhibitors. Briefly, exponentially growing MV4-11 cells were seeded into 6-well microplates at concentration of 1×10^6^ cells per well and were treated with control or sunitinib at the concentrations of 2×IC50, 4×IC50 and 8×IC50 or crotonoside at the concentrations of 2×IC50 (13.8 nM), 4×IC50 and 8×IC50 or in combinations of 2×IC50, 4×IC50, 8×IC50 sunitinib and 2×IC50 HPOB, 2×IC50, 4×IC50, 8×IC50 sunitinib and 2×IC50 HPOB, 2×IC50 RGFP966 and 2×IC50, 4×IC50, 8×IC50 sunitinib, and 2×IC50 HPOB, 2×IC50 RGFP966 and their combination. After 24 h treatment, cells were counted, harvested, washed with PBS and incubated in novel 6-well microplates with fresh culture medium. Cells number was counted daily for 6 days. The cell growth curve was constructed and the post-inhibition effects of these inhibitors were analyzed. As shown in Figure 3, MV4-11 cells treated with sunitinib exhibited inhibition for a short duration and resumed growth after incubated in fresh culture medium. However, MV4-11 cells treated with 2×IC50 crotonoside resumed the growth slowly after replenishment with fresh medium and this effect significantly increased in a dose-dependent manner, with increasing concentrations, increased inhibition was observed for treatments with 4 or 8×IC50 of crotonoside. MV4-11 cells treated with the different combinations also resumed growth slower than the cells treated with inhibitor alone. It is noteworthy that MV4-11 cells treated by the combination of HPOB, RGFP966, and sunitinib exhibited slower growth than other combinations and even no growth was observed in cells treated by 4 or 8×IC50 of crotonoside. These findings suggest that crotonoside exhibit more efficient selective inhibition in AML cells via inhibition of FLT3 and HDAC3/6.

### Influence of FL or IL-3 on the anti-AML activity of crotonoside, RGFP966, HPOB and Sunitinib

**Figure 4:**
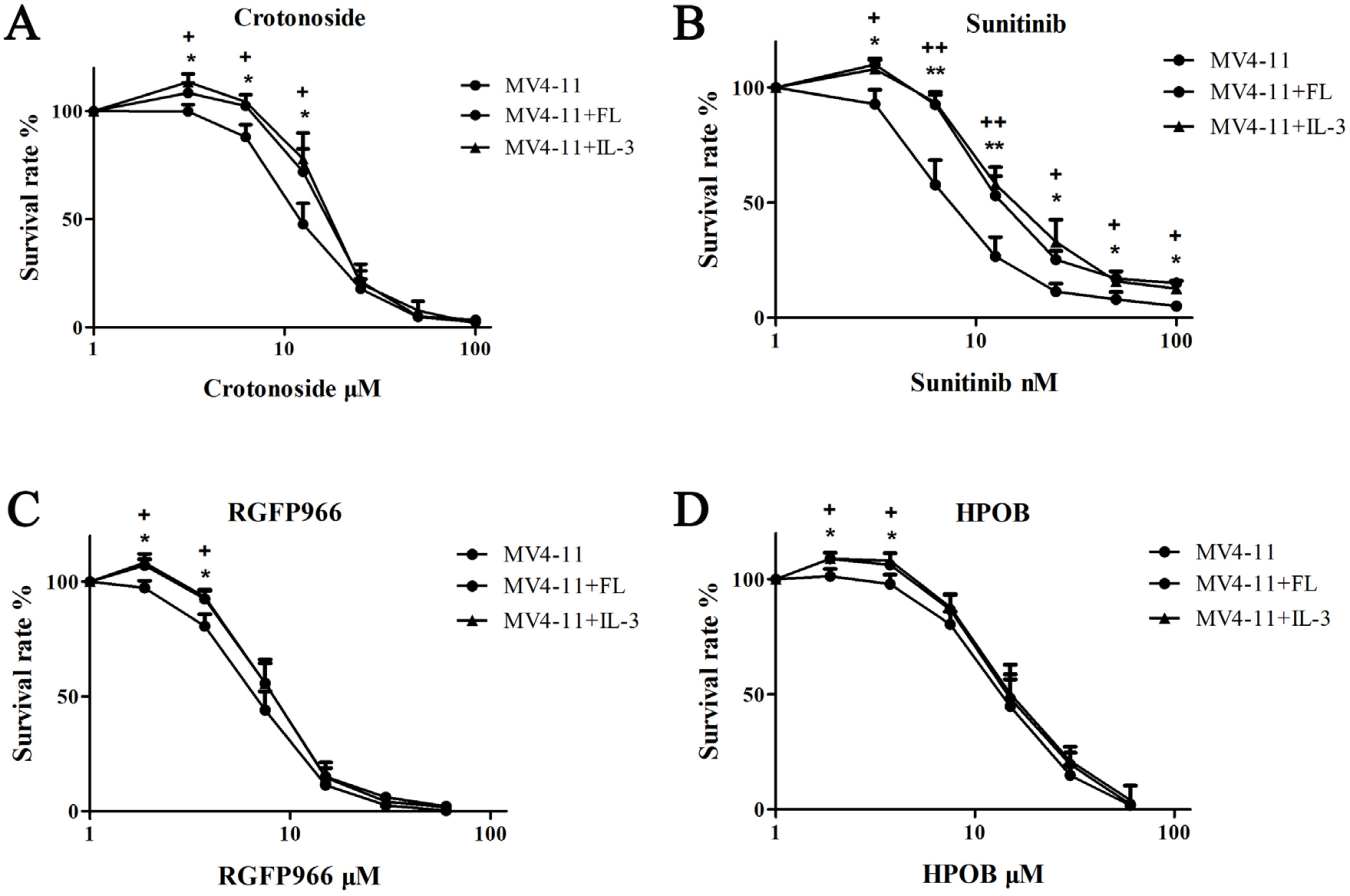
Effect of FL or IL-3 on the anti-AML activity of crotonoside The anti-AML activity of crotonoside, sunitinib, RGFP966, and HPOB was evaluated in the MV4-11 cells cultured with 100 ng/ml FL or 50 ng/ml IL-3. **(A)** FL or IL-3 induced the activity of crotonoside at the effective concentrated of 12.5 μM. **(B)** FL or IL-3 reduced the inhibitory activity of sunitinib at all effective concentrations of 12.5, 25, 50, 100 nM (FL: ^*^, *p*<0.05; ^**^, *p*<0.01; IL-3: +, *p*<0.05; ++, *p*<0.01). **(C** and **D)** FL or IL-3 had no significant influence on the inhibition of HDAC3 or HDAC6 inhibitors at the effective concentrations.

Next, we also evaluated the addition of exogenous FL or IL-3 in the MV4-11 cells to determine the significance of crotonoside in comparison to classic FLT3 inhibitors. As shown in Figure 4, the addition of FL or IL-3 significantly reduced the inhibitory activity of sunitinib at all effective concentrations of 12.5, 25, 50, 100 nM (*p*<0.05), and influenced the activity of crotonoside at concentrations of 6.25 and 12.5 μM (*p*<0.05). However, the additions of FL or IL-3 not significantly influence the inhibition effect of HDAC3 or HDAC6 inhibitor in the effective concentrations. thus, the inhibitory effect of crotonoside at concentrations lower than 12.5 μM is mainly mediated through direct inhibition of FLT3. Meanwhile, crotonoside could reverse the influence of FL or IL-3 atconcentrations higher than 25 μM via the suppression of expression of HDAC3 and HDAC6.

### Cell cycle and apoptosis assays

**Figure 5:**
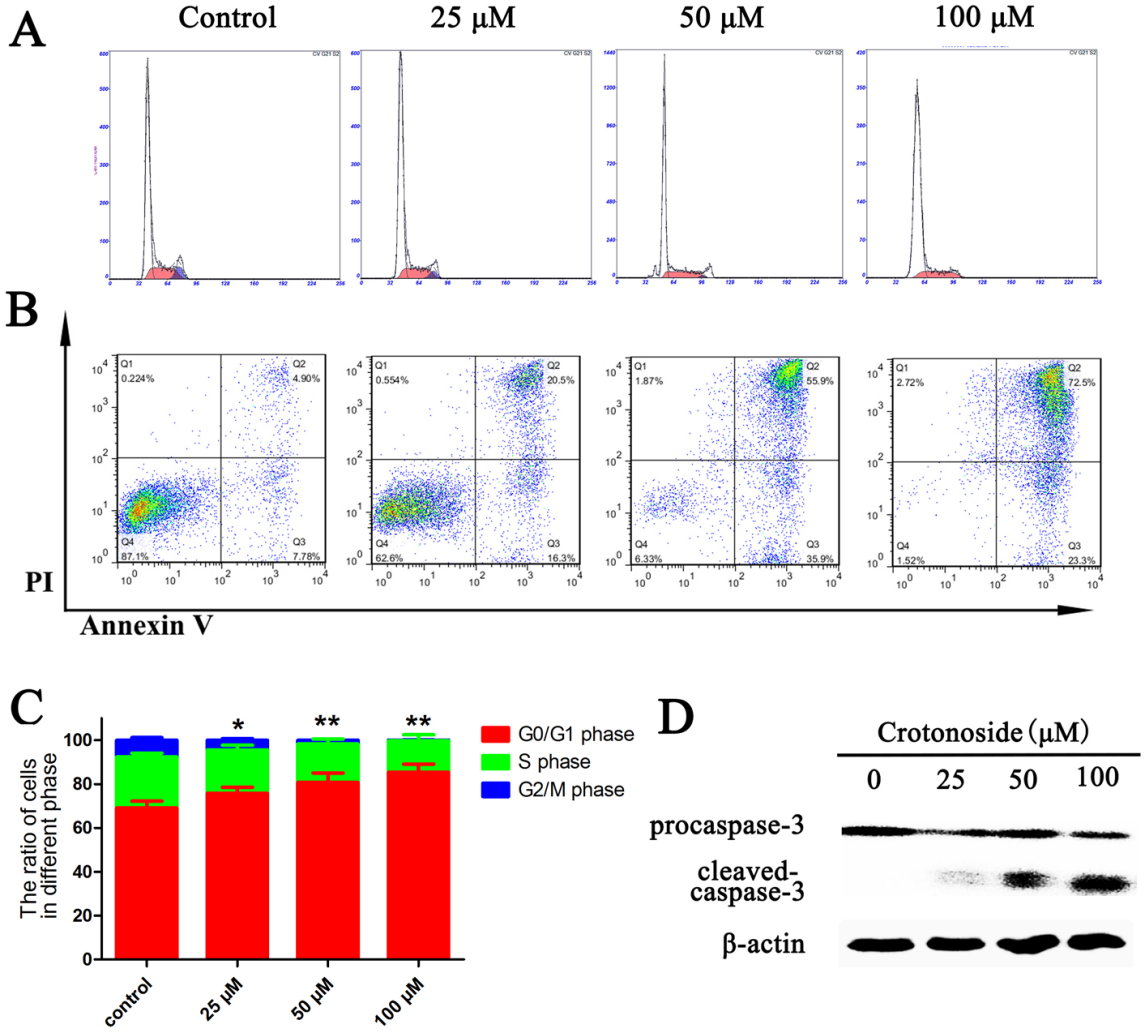
Crotonoside induced cell cycle arrest and apoptosis in AML cells **(A** and **C)**, MV4-11 cells were treated with different concentrations of crotonoside or 0.1% DMSO for 12 h. The cell cycle was assessed by propidium iodide staining and flow cytometry. The ratio of MV4-11 cells in G0/G1 phase increased significantly, while the ratio of cells in S and G2/M phase significantly decreased (^*^, *p*<0.05; ^**^, *p*<0.01). **(B)** MV4-11 cells treated with crotonoside or 0.1% DMSO for 24 h and the apoptotic cells were defined by Annexin-V and PI co-staining. The early and late apoptotic cells are shown in the right lower and right upper quadrant, respectively. **(D)** The cells lysate of MV4-11 cells treated with crotonoside for 24 hours was also analyzed using a caspase-3 antibody, and the results show a significant dose-dependent decrease in pro–caspase-3 levels and a dose-dependent increase in cleaved caspase-3 levels with increasing concentrations of crotonoside.

Cell cycle analysis by PI staining and a flow cytometry were performed 12 hours after treatment of MV4-11 cells with a series of concentrations of crotonoside. MV4-11 cells exhibited a dose-dependent increase in the percentage of G0/G1 phase and a dose-dependent decrease in the percentage of G2/M and S phases cells (Figure 5A and 5C). These results thus suggest that the effect of the crotonoside on the MV4-11 cells appears to be due to cell cycle arrest in G0/G1. Two-color flow cytometry analyses were performed using AnnexinV/PI staining, we found that the percentage apoptosis was slightly higher when MV4-11 cells were treated with different concentrations of crotonoside (25, 50, and 100 μM). Treatment of cells for 24 h with crotonoside led to concentration-dependent changes in the number of apoptotic MV4-11 cells, and a concentration of 50 μM crotonoside induced late apoptosis in about 50% AML cells (Figure 5B). The level of cleaved caspase-3 was also measured. After 20 h of treatment with different concentrations of crotonoside, MV4-11 cells were lysed and analyzed using Western blot assay. As shown in Figure 5D, a dose-dependent decrease in the level of pro-caspase-3 and a dose-dependent increase in the level of the cleaved caspase-3 fragments were observed for crotonoside-treated MV4-11 cells at the concentration of 25∼100 μM. These results indicated that crotonoside can induce cell cycle arrest in G0/G1 and cause apoptosis at concentrations above the IC50 value in MV4-11 cells.

### *In vivo* effects of crotonoside against MV4-11 tumor xenografts

**Figure 6:**
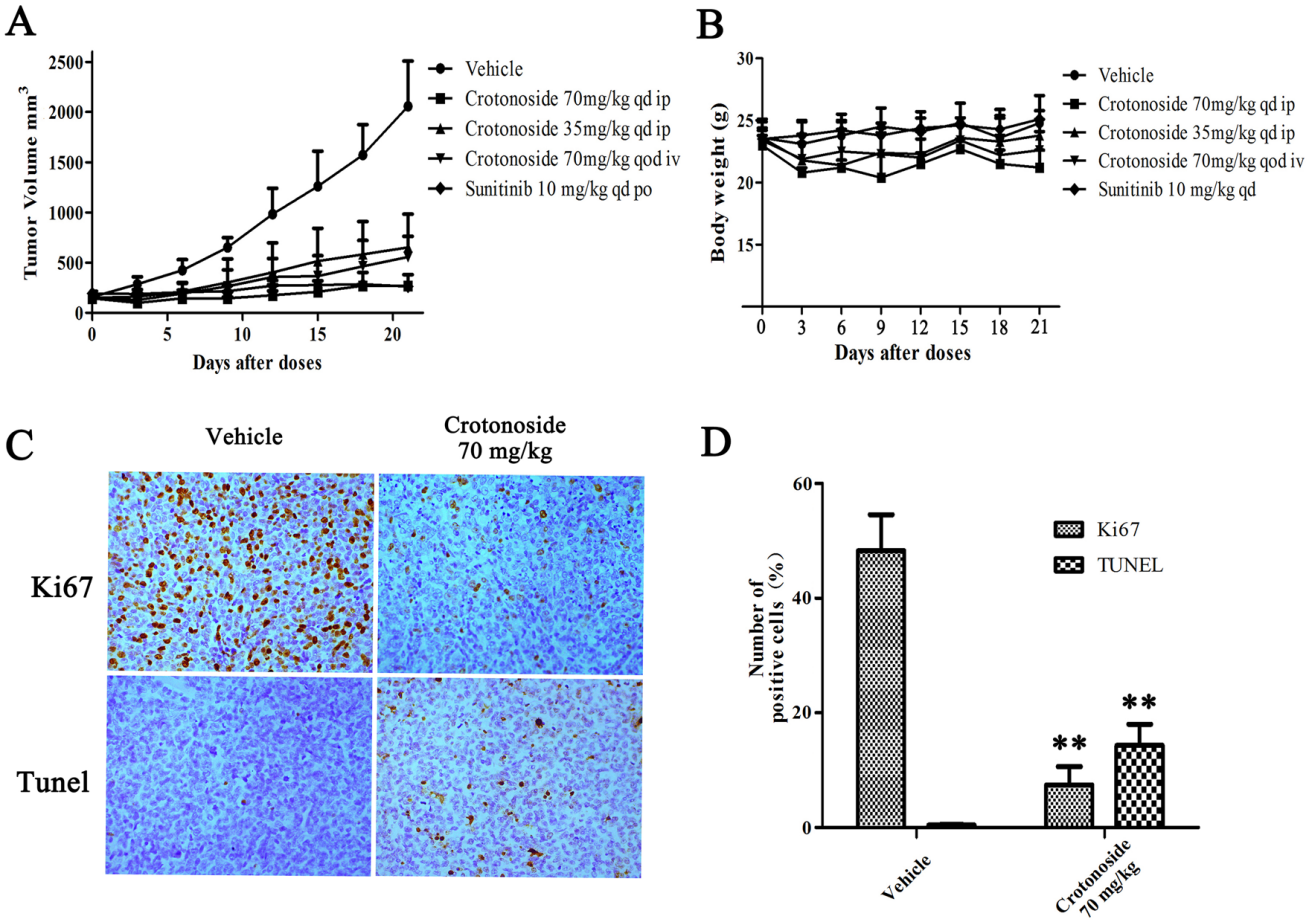
*In vivo* effects of crotonoside against s.c. MV4-11 tumor xenografts **(A)** MV4-11 cells (1×10^7^/mouse) were s.c. injected into NOD-SCID mice, and treatment with crotonoside was initiated when the tumors grew to 150∼300 mm^3^. Crotonoside significantly inhibited the HCT116 tumor growth at the dose of 70 or 35 mg/kg/d, and sunitinib as positive control exhibited marked anti-tumor activity. **(B)** the mice body weights among the groups were analyzed after 21 days of treatment. The mean values ± SD are shown in **(C** and **D)** Following the treatment of crotonoside at indicated time points, the MV4-11 tumors were collected (three per group). Ki67 and TUNEL assays show that crotonoside significantly inhibited the proliferation and induced apoptosis in the MV4-11 cells *in vivo* (^*^, *p*<0.05; ^**^, *p*<0.01).

The *in vivo* antitumor activity of crotonoside was evaluated in the subcutaneous tumor xenograft model of NOD-SCID mice. The animals were treated with different doses of crotonoside at 70 mg/kg/day (i.p.), 35 mg/kg/day (i.p.) and 70 mg/kg (qod, i.v.) or with vehicle (i.p.) alone. Sunitinib at a dose of 10 mg/kg/day (po) was considered as a positive control. Treatment of cells for 24 h with crotonoside induced a significant antitumor activity and inhibited xenograft tumor progress as compared to treatment with vehicle. Treatment with crotonoside at a dose of 70 mg/kg/day (i.p.) and treatment with sunitinib at 10 mg/kg/day (po) exhibited the highest tumor inhibition rates of 93.5% and 96.3%, respectively (Figure 6A). Treatment with crotonoside at doses 35 mg/kg/day (i.p.) and 70 mg/kg (qod, i.v.) showed the tumor inhibition rates of 73.6% and 78.3%, respectively. The body weight of mice was monitored once every 3 days through the whole course of the experiment. As illustrated in Figure 6B, treatment with crotonoside at doses of 70 mg/kg/day (i.p.) and 70 mg/kg (qod, i.v.) produced a slight loss in body weight (less than 20%) during the first 6 days of treatment, and exhibited no further loss on the last day of the treatment. No significant effects in the gross measurements such as skin ulcerations or lethality were observed in crotonoside group.

To further characterize the *in vivo* antitumor activity of crotonoside, comparative histopathological analyses of tumor tissues resected from the vehicle-treated and crotonoside-treated animals were performed. As shown in Figure 6C, tissue sections from the vehicle-treated tumors stained strongly with Ki67, indicating a tumor with a high proliferation index. Conversely, tissue sections from the tumors treated with 70 mg/kg/day (i.p.) of crotonoside showed a marked reduction in Ki67-positive cells compared to the control group. Furthermore, we analyzed the apoptosis induced by crotonoside *in vivo* using TUNEL assay. As observed from Figure 6D, a significantly higher percentage of TUNEL-positive cells were observed in the crotonoside-treated group as compared to vehicle-treated control group, suggesting that crotonoside induced apoptosis in tumor cells (*p*<0.05).

## DISCUSSION

Acute myeloid leukemia (AML) is the most common and complex form of acute leukemia, with an incidence that increases with advanced age [36]. Although the mortality has decreased significantly in the past 4 decades with the treatment of new chemotherapeutics and their combined treatment, recurrence and drug resistance frequently occurs within a short time [37]. Targeted therapies, especially the use of FLT3 inhibitor, have shown promising results in recent years, but the considerable genetic, epigenetic, phenotypic heterogeneity and multiple mutations in AML cells led to the drug resistance [6, 7, 20]. Therefore, we hypothesized that agents simultaneously inhibiting more than one targets that selectively promote the AML progression might bring an improved clinical outcome in the treatment of AML.

Traditional Chinese drug therapies have exhibited therapeutic effect in the treatment of AML. The treatment using As2O3 is an effective and relatively safe drug in APL patients refractory to ATRA and conventional chemotherapy [38, 39]. Plants are the main source of traditional Chinese drugs, and about 50% of modern drugs in clinical use are also natural plant products, many of which have the ability to control tumor cell [40]. The seed of *Croton tiglium L.*(family Euphorbiaceae), well known as “Badou” in China, is a traditional Chinese medicinal herb that widely used to treat gastrointestinal disorders, intestinal inflammation, and rheumatism in traditional Chinese medicine [41]. Crotonoside is one of the major constituent of “Badou”, but has been rarely reported about its bioactivity for medicinal value [42]. Our study was performed to evaluate the anti-tumor activity of crotonoside and found that crotonoside could inhibit all of FLT3 and HDAC3/6, and exhibited selective inhibition pattern toward AML cells.

*in vitro*, crotonoside exhibited potent anti-proliferative activities against FLT3-driven AML cell line MV4-11, and exhibited low toxic effects in normal cells and other tumor cell lines. *in vivo*, crotonoside significantly inhibited the growth of MV4-11 xenograft tumor, and reduced the tumor volume significantly with well toleration. Immunoblot detection indicated that crotonoside at concentrations higher than 12.5 μM could inhibit the phosphorylation of FLT3 and its downstream signaling proteins Erk1/2, Akt/mTOR and STAT5 in 7 hours, without modulating the expression of these proteins. Further, crotonoside was found to significantly inhibit the expression of FLT3 after a 20 h treatment at concentrations higher than 25 μM. In addition, crotonoside inhibited both transcription factors’ expression of NF-κB-p65 and c-Myc, but the selective FLT3 inhibitor sunitinib only inhibited the expression of c-Myc. These results indicated that crotonoside was able to inhibit not only FLT3 but also other targets that may selectively promote the AML progression.

HDACs play a key role in regulating gene expression by changing chromatin structure, and V Novotny-Diermayr have demonstrated that the pan-HDACs inhibitor SB939 could inhibit the expression of FLT3 in AML cells [43]. We performed western blot assay and proved that crotonoside significantly inhibited the expression of HDAC3 and HDAC6 without modulating the expression of other isoforms, such as HDAC1, HDAC2 and HDAC4. Further, we found that selective HDAC3 inhibitor could inhibit the expression of FLT3 and NF-κB-p65, and exhibit selective inhibition activity pattern toward AML cells. Meanwhile, we found that HDAC6 inhibitor also exhibit selective inhibition activity pattern toward AML cells by inhibiting the expression of c-Myc. Interestingly, AML cells raised the expression of HDAC6 after being treated by the classic FLT3 inhibitor sunitinib, which also have the inhibition activity in the expression of c-Myc. These data indicated that HDAC3/NF-κB-p65 and HDAC6/c-Myc were novel signaling pathways beside FLT3/c-Myc selectively promoting the AML progression and contributing to the transformed phenotype of AML.

Selective inhibition of FLT3, HDAC3 or HDAC6 have been proved useful for the treatment of acute myeloid leukemias, but the benefits of combinations of HDAC3, HDAC6 and FLT3 inhibitors were rarely reported. We evaluated the post-inhibition effect of every single inhibitor and the combinations among the selective inhibitors to analyzed the the advantages of crotonoside by inhibiting all of the three targets. Our results showed that combinations of any two inhibitors have more efficient inhibition activity than every single inhibitor, and the combination of all of HDAC3, HDAC6 and FLT3 inhibitors exhibited the most efficient inhibition activity especially. An old Chinese saying goes “ye chang meng duo”, means “A long delay means trouble”. For tumor cells, the long survival time means the more chances for the activation of additional signaling pathways beside the FLT3. The “post-treatment” effects indicated that crotonoside could kill the AML cells in 24 hours, and sunitinib only inhibited the growth of AML cells without inhibiting their survival at higher concentrations. Finally, our study indicated that crotonoside exhibit more efficient inhibition pattern toward AML cells via simultaneous targeting the FLT3 and decreasing the expression of HDAC3/6.

Recently, single cell analysis demonstrated that not all of the leukemia cells in genomic FLT3-ITD+ patient possess the FLT3-ITD mutation [34, 35]. Thus, the intra-tumor heterogeneity and multiple mutations are important contributors in the relapse and FLT3 inhibitor resistance. In our study, we also detected the growth inhibitory activity of crotonoside and HDAC3/6 inhibitor against KG-1, a general AML cell line with no FLT3 wild-type or FLT3-ITD mutation. As shown in results, crotonoside exhibited selective inhibition activity in KG-1 cells with IC50 values of 17.2±4.6 μM, and could decrease the expression of HDAC3 and HDAC6 without modulating the expression of other isoforms, such as HDAC1, HDAC2 and HDAC4. Meanwhile, RGFP966 and HPOB also selectively inhibited the viability of KG-1 with IC50 values of 6.91±2.2 μM and 21.49±7.7 μM. Therefore, crotonoside also could inhibit the AML cells with other genetic backgrounds via inhibiting the expression of HDAC3/6.

## CONCLUSION

To sum up, crotonoside has the ability to block all of the FLT3, HDAC3 and HDAC6 signaling in AML cells. This compound exhibited selective and more efficient inhibition pattern toward AML cells *in vitro* and significantly inhibit the AML xenograft tumor growth *in vivo*. Collectively, crotonoside could be a promising new lead compound for the treatment of AML, and inhibitors targeting all of the FLT3, HDAC3 and HDAC6 will provide more effective treatment strategy. However, crotonoside might have off target effects in the inhibition of HDAC3 and HDAC6's expression. more mechanistic and pre-clinical studies are still necessary before considering clinical trials.

## MATERIALS AND METHODS

### Materials

For the present study, 3-(4,5-dimethylthiazol-2-yl)-2,5-diphenyltetrazolium (MTT), dimethyl sulfoxide (DMSO), Hoechst stain, and propidium iodide (PI) were procured from Sigma Chemical Co. (St. Louis, MO). The primary antibodies to FLT3, STAT5, ERK_1/2_, Akt caspase-3, β-actin, HDACs were acquired from Cell Signaling Technology (Beverly, MA). Annexin-V-FITC/PI Apoptosis Detection Kit and BCA protein assay kit were purchased from Beyotime (Shanghai, China). Crotonoside were obtained from the Must Biotech Co., Ltd. (Chengdu, China). All the chemicals used in the study were of analytical and culture grade.

### Cell culture

The cell lines were obtained from the American Type Culture Collection (Manassas, VA, USA). MOLM-13 cells were donated by professor Shengyong Yang (Sichuan University). All the cells except for MV4-11 cells were grown in RPMI 1640 or DMEM culture medium supplemented with 10% fetal bovine serum (v/v). MV4-11 cells were cultured in the IMDM culture medium. All cultures were maintained at 37°C and 5% CO_2_.

### Cell viability assay

Cell viability was assessed using the MTT colorimetric assay. Briefly, exponentially growing tumor cells were harvested and seeded into 96-well microplates. After overnight incubation, cells were treated with equal volume of medium containing various concentrations of crotonoside ranging from 3.12 μM to 200.0 μM. After the incubation period (72 hours at 37°C), 20μL of 5 mg/mL MTT reagent was added per well and incubated for 2∼4-hour, and 50 μL of 20% acidified SDS per well was then added to lyse the cells. The absorbance was measured at 570 nm with a SpectraMAX M5 microplate reader (Molecular Devices). All experiments were performed in triplicate. The relative cell viability (%) was expressed as a percentage relative to the control cells treated with DMSO (0.1%).

### Cell cycle and apoptosis assays

The cell cycle and apoptosis assays were performed using Flow cytometry. Briefly, MV4-11 cells were exposed to increasing concentrations of crotonoside for 16 or 24 hours. After treatment MV4-11 cells were harvested, washed with PBS and stained for apoptosis using the Annexin V-fluorescein isothiocyanate (FITC) Apoptosis Detection Kit. Samples were analyzed by flow cytometry using cell Quest software (BC Epics XL, MIAMI, Florida, USA).

For cell cycle assays, cells were harvested with 0.05% trypsin and stained with hypotonic PI staining solution (50 mg/mL PI) containing 0.1% Triton X-100 in 0.1% sodium citrate solution and RNase A. The cell cycle analysis of nuclei suspension was immediately analyzed using flow cytometry (Coulter Episs XL, Beckman Coulter).

### Western blot analysis

MV4-11 and KG-1 cells were incubated for 8 or 20 hours in medium containing gradient concentration of crotonoside or other agents. Cells were harvested, washed and the total proteins were extracted in ice-cold RIPA buffer (10 mM Tris-HCl (pH 7.8), 1% NP40, 0.15 M NaCl, 1 mM EDTA, 10 μM aprotinin, 1 mM NaF and 1 mM Na3VO4). Protein concentrations were quantified using BCA Protein Assay Kit. For western blot assay, equal proteins were separated by 10% -15% SDS-PAGE, transferred onto PVDF membranes and probed with various primary antibodies (Cell Signaling Technology) overnight at 4 C. Antibodies included; FLT3, Phospho-FLT3, STAT5, Phospho-STAT5, ERK_1/2_, Phospho-ERK_1/2_, Akt, Phospho-Akt, caspase-3, β-actin, HDAC1, HDAC2, HDAC3, HDAC4, HDAC6. The membranes were washed three times with TBST and incubated with horseradish peroxidase (HRP)-conjugated secondary antibody. Specific antibody binding was detected by chemiluminescent kit on Kodak X-ray films.

### Effect of crotonoside on Tumor Growth *in vivo*

Six- to seven-week-old female NOD-SCID mice (Beijing HFK Bioscience) were used and housed in a sterile environment and fed a standard diet *ad libitum*. MV4-11 cells were harvested during the exponential growth phase. For subcutaneous injection, single cell suspensions (1 × 10^8^ cells/ml) in serum-free medium were injected into the hind flank at 100μl/site to establish the xenograft model. When the tumors grew to a size of ∼200 mm^3^, The mice bearing tumors were equally divided into different groups (6 mice each group) and received intraperitoneal (i.p) and intravenous (i.v.) injection of crotonoside (70 mg/kg/day i.p., 35 mg/kg/day i.p. and 70 mg/kg pod i.v., respectively) or vehicle (2% DMSO+ 98% normal saline) for 21 days. Positive control group mice were injected with sunitinib (20 mg/kg/day). Tumor growth and body weight were measured every 3 days with Vernier calipers during the treatment *in vivo*. The tumor volume was calculated as follows: tumor volume = a×b^2^/2 (a, longest diameter (length); b, shortest diameter (width)).

### Immunohistochemical analysis

To investigate whether crotonoside inhibited the tumor growth *in vivo*, the immunohistochemical stain was performed using Ki 67 antibody (Thermo Fisher Scientific, CA). All mice were sacrificed when the tumors reached approximately 200 mm^3^. Tumors were harvested, fixed and paraffin embedded. For immunohistochemical analysis of Ki-67, tumor tissue sections (4∼8 μm) were prepared and subjected to immunostaining with a Ki67 antibody. In addition, Apoptosis was detected by transferase-mediated dUTP nick-end labeling (TUNEL) assay (Roche Applied Science) as described by the manufacturer. Apoptotic cells were then visualized with an Olympus digital camera attached to a light microscope.

### Statistical analysis

All the *in vitro* experiments were performed in triplicate. Numerical data are expressed as mean ± SD. Statistical analysis was performed by. The value p and data were expressed as mean± SD or mean± SEM. Statistical analysis was performed by analysis of One-way ANOVA and/or Student's t-test for independent analysis using Graph-Pad Prism 6.0. The value p <0.05 was considered statistically significant when P <0.05.
